# Comparison of artificial intelligence algorithms and their ranking for the prediction of genetic merit in sheep

**DOI:** 10.1038/s41598-022-23499-w

**Published:** 2022-11-04

**Authors:** Ambreen Hamadani, Nazir A. Ganai, Syed Mudasir, Syed Shanaz, Safeer Alam, Ishraq Hussain

**Affiliations:** Sher-e-Kashmir University of Agricultural Sciences and Technology of Kashmir, Srinagar, India

**Keywords:** Animal breeding, Machine learning

## Abstract

As the amount of data on farms grows, it is important to evaluate the potential of artificial intelligence for making farming predictions. Considering all this, this study was undertaken to evaluate various machine learning (ML) algorithms using 52-year data for sheep. Data preparation was done before analysis. Breeding values were estimated using Best Linear Unbiased Prediction. 12 ML algorithms were evaluated for their ability to predict the breeding values. The variance inflation factor for all features selected through principal component analysis (PCA) was 1. The correlation coefficients between true and predicted values for artificial neural networks, Bayesian ridge regression, classification and regression trees, gradient boosting algorithm, K nearest neighbours, multivariate adaptive regression splines (MARS) algorithm, polynomial regression, principal component regression (PCR), random forests, support vector machines, XGBoost algorithm were 0.852, 0.742, 0.869, 0.915, 0.781, 0.746, 0.742, 0.746, 0.917, 0.777, 0.915 respectively for breeding value prediction. Random forests had the highest correlation coefficients. Among the prediction equations generated using OLS, the highest coefficient of determination was 0.569. A total of 12 machine learning models were developed from the prediction of breeding values in sheep in the present study. It may be said that machine learning techniques can perform predictions with reasonable accuracies and can thus be viable alternatives to conventional strategies for breeding value prediction.

## Introduction

The fundamental responsibility of an animal breeder is to ensure that the animals of each generation are better in performance than the previous generation. This is achieved through accurate identification of superior animals and their scientific selection which in turn depends on the prediction of breeding values. The process of computation of the genetic merit of animals is largely data-driven and requires complex computations. Techniques developed by breeders and statisticians have worked very well so far and have yielded tremendous results for improving production. However, the fast-evolving world is now facing new, hitherto unknown challenges like population explosion, climate change, and environmental degradation only to name a few. In response, farming practices are evolving, and new technologies are being adopted. All this is leading to the generation of an enormous amount of diverse data daily and age-old methods and conventional strategies are unable to keep up with this growing amount of data and they alone cannot suffice in meeting the challenge of managing the data quickly and accurately. Among the methodologies used for the prediction of breeding values, Best Linear Unbiased Prediction (BLUP) is considered to be the most accurate as it combines all this information optimally and automatically^1^. breeding value estimations are cumbersome and extremely difficult for people with little know-how of animal breeding. Therefore, if the technique is performed only once and the labels are subsequently used for training a model, that model can be deployed and used multiple times without any burden on computational resources.

State-of-the-art machine learning techniques for data mining like neural networks, decision trees, etc. in animal genetics and breeding may become major game-changers in this regard. These technologies are already chauffeuring the world towards a major technological revolution. Data-driven intelligent systems as well as cutting-edge digital fabrication technologies are already rapidly becoming a part of the biological world and are making it possible to embrace new and innovative methods to deliver food security, economic opportunities, and of course environmental sustainability. They can, therefore, transform the science of animal breeding which, in itself, is a data-intensive science.

The fact that animal genetics and breeding (AGB) is based on biometrical genetics and advanced statistics which is also the core of artificial intelligence is yet another reason to integrate the two. Also, the central paradigm of animal breeding revolves around making futuristic predictions which is also the heart of artificial intelligence. On top of this, data mining techniques offer a myriad of other advantages as well; they are rapid, low cost, accurate and can also handle nonlinear and complex data even when it is imprecise and noisy^2^ which is not possible using conventional techniques.

## Related work

Artificial Intelligence has been transforming various spheres of life for quite some time now. For example, research is being conducted for the prevention and control of COVID-19^3^, for the reduction in the emission of greenhouse gases^4^, and their impact on climate predictions^5^, etc. The interdisciplinary work combining Artificial Intelligence and Machine Learning in Animal Sciences is picking up the world over^2,6–8^. Research in this area has been done by various researchers (Table 1). Though the field of machine learning has the potential to revamp every sphere of animal sciences, this field is still in its infancy. The studies reported in this review show great promise of machine learning in improving animal sciences, the number of studies that are specific to animal genetics and breeding is even more insufficient to explore and unleash the full potential of machine learning for animal genetic improvement.

**Table 1:** A brief review of the use of various algorithms in animal sciences.

Algorithm(s)	Prediction of	Reference
Multivariate adaptive regression splines (MARS)	Body weights	^40^
Radial basis function (RBF)	Body weights	^40^
Multivariate adaptive regression splines (MARS)	Fattening weights	^38^
Multiple regression	Live body weights	^61^
Multilayer perceptron	Body weights	^39^
Convolutional neural networks (CNNs)	Feed intake and milk production measurement and frequency	^62^
Genetic algorithms	Problems associated with low-birth-weight infants	^42^
Support vector regression	Body weights	^63^
Regression trees	Body weights	^64^
Convolutional neural networks (CNNs)	Feed intake and milk production measurement and frequency	^62^
CNN's using RGB—D cameras	Feed intake for individual cows	^62^
Machine vision-based visual image analysis	Monitor BW in growing pigs for feeding	^65^

A comprehensive study comparing the important and state-of-the-art supervised machine learning techniques for the prediction of breeding values of animals could not be traced. This study is therefore novel research to explore artificial intelligence techniques in depth so that not only would the potential of each technique be explored but the best algorithms could be chosen for use on the farms. Through this study, reusable machine learning models could also be created which, upon deployment on servers could be used by farmers for genetic improvement of their animals. This would particularly be useful in developing parts of the world like India where the scientific selection of animals is rare, and selection is mostly intuition-based. The present study was therefore undertaken to fill such gaps between these two critical subjects, viz. animal breeding, genetics, and artificial intelligence.

## Results

### Missing values

The number of missing features for the dataset was low. The lighter colors in the figure represent missing values. Our results indicate that out of the numerical variables in the data, birthweights had the least number of null values.

### VIF and feature selection

The VIF results for the dataset indicated that most of the variables were lowly or moderately correlated with most of the features having variance inflation factors of less than 3. The sire breeding values, and dam breeding values had high feature selection scores, but they were not used for training the model.

### Input variables

The features/input variables selected for the machine learning approaches included birth weight (BW), weaning weight (wean), 6-month weight (m6), 9-month weight m9, 12-month weight (12mwt), sire 12-month weight (sire12mwt), dam 12-month weight (dam12mwt), sex effect, year effect. These were done based on feature selection based on the selection score (> 10).

### Machine learning algorithms

#### PCR

The results of the principal component analysis indicated that a total of 7 variables explained greater than 95% variance. The explained variance ratios were 0.39, 0.12, 0.12, 0.11, 0.10, 0.07, 0.05 for the extracted features. For the principal component regression (PCR), the validation dataset was heuristically set at 10%. The variance inflation factors for all features were 1.

#### OLS

Ordinary Least Squares (OLS) The prediction equations based on their statistical significance are given in Table 2. The overall adjusted R^2^ value of the prediction equation was highest for the feature-selected dataset with a higher number of features. Equations (3a) and (4a) given in Table 2 contain all variables in the dataset for the dataset upon which PCA and feature selection was performed respectively. Equations (3b) and (4b) contain only the features which were found to be highly significant in Eqs. (3a) and (4a) respectively.

**Table 2:** Prediction equations for PCA features based on the significance.

No	Prediction equation for PCA features	R^2^
3a	− 0.415 + 0.292 × 1 − 0.341 × 2 − 0.221 × 3 + 0.037 × 4 − 0.178 × 5 − 0.076 × 6	0.514*
3b	− 0.415 + 0.292 × 1 − 0.341 × 2 − 0.221 × 3–0.178 × 5–0.076 × 6	0.513*

No	Prediction equation for feature selected features	R^2^
4a	− 0.4152 − 0.013bw − 0.0218wean − 0.053m6 − 0.168m9 − 0.223m12 − 0.080 × 6sirem12 + 0.324damm12 − 0.002sexe + 0.516 × 9yeare	0.569*
4b	− 0.415 − 0.109m9 − 0.206m12 − 0.078sire12m + 0.323dam12m + 0.525yeare	0.568*

Where birthweight = bw, weaning weight = wean, 6-month weight = m6, 9-month weight = m9, 12-month weight = m12, sire's 12-month weight = sire12m, dam's12 month weight = dam12m, sex effect = sexe, year effect = yeare.

#### Bayesian ridge regression

**Figure 1:**
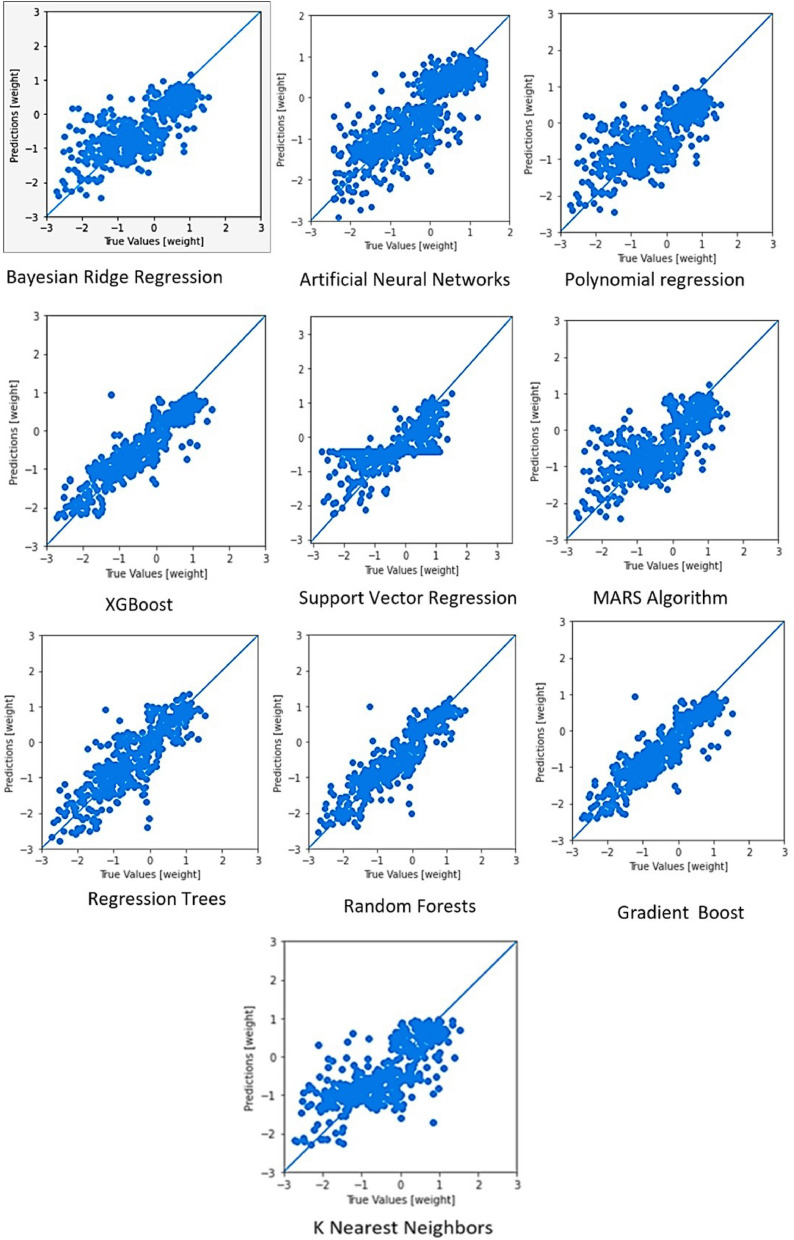
Correlation between true and predicted values for breeding value prediction.

The results of the Bayesian regression model training for the dataset are given in Table 3. The correlation between true and predicted values for the algorithms for breeding value prediction is given in Fig. 1.

**Table 3:** Model comparison for PCR and Bayesian ridge regression.

Measure	PCR		Bayesian ridge regression (test)
	Mean cross-validation	Mean test values	
Mean absolute error	0.510	0.495 s	0.498
Root mean squared error	0.718	0.644	0.648
Coefficient of determination	0.440	0.552	0.547
Correlation coefficient	0.556	0.746	0.742

#### Artificial neural networks

A total of 35 models were trained to determine which group of hyperparameters could train the model best. Out of the models trained the top 13 are given in Table 4. For hyperparameter optimization using *axclient*, with the increase in the number of iterations, the correlation coefficient also increased. However, the results obtained did not improve significantly after the 1000th trial.

**Table 4:** Artificial neural networks for prediction of breeding values.

	Heuristically trained models													Hyperparameter Optimization		
	1	2	3	4	5	6	7	8	9	10	11	12	13	Model1	Model2	Model3
**Optimum hyperparameters**																
Activation	swish	ReLU	swish	ReLU	ReLU	tanh	swish	Leaky ReLU	PReLU	swish	swish	swish	swish	ReLU	ReLU	ReLU
Layers	15	6	3	4	7	7	15	3	15	7	13	11	12	9	9	9
Neurons	90	90	90	20	50	80	90	90	90	80 (3)	80 (5)	80 (3)	80 (3)	257	40	40
										10 (4)	10 (8)	10 (8)	10 (8)			
Optimizer	adam	adam	adam	adam	adam	adam	adam	adam	adam	adam	adam	adam	rAdam	adam	adam	adam
Learning rate	0.0009	9E-04	9E-04	9E-04	9E-04	9E-04	9E-04	9E-04	0.0009	0	1E-04	9E-04	1E-05	0.005	0.002	0.002
Batch size	20	20	20	20	20	20	20	20	20	50	10	100	100	10	30	30
Epochs (500)	31	17	32	31	26	12	31	30	13	16	56	58	58	12/17	17/17	17/17
**Evaluation metrics**																
Validation MSE	0.276	0.312	0.254	0.32	0.287	0.502	0.276	0.27	0.363	0.29	0.291	0.252	0.284	0.574	0.72	0.631
Validation MAE	0.4	0.421	0.387	0.441	0.413	0.574	0.4	0.397	0.481	0.42	0.416	0.374	0.399	0.6	0.694	0.647
Test MAE	0.37	0.39	0.374	0.418	0.395	0.573	0.372	0.387	0.477	0.4	0.391	0.359	0.385	0.614	0.606	0.582
Test MSE	0.244	0.28	0.248	0.298	0.276	0.519	0.244	0.264	0.367	0.29	0.261	0.231	0.266	0.576	0.541	0.488
Test loss	0.244	0.28	0.248	0.298	0.276	0.519	0.244	0.264	0.367	0.29	0.261	0.231	0.266	0.576	0.541	0.488
Test r	0.841	0.825	0.838	0.806	0.821	0.621	0.841	0.825	0.75	0.81	0.828	0.852	0.836	0.569	0.675	0.734
RMSE	0.494	0.53	0.498	0.546	0.525	0.72	0.494	0.514	0.589	0.53	0.511	0.481	0.516	0.759	0.675	0.699
Iterations														500	1000	2000

#### Support vector machines

The algorithm with default parameters was able to predict the test labels with a higher correlation than the grid search algorithm.

#### Regression trees and random forests

Random search algorithm showed the most model convergence. Random forests outperformed regression trees in terms of all the scoring criteria used in the present study. The coefficients of determination for regression trees were 0.86 and for random forests (grid search and random search), they were 0.905 and 0.904 respectively.

#### Gradient boost

The correlation obtained by grid search was slightly lower than using the algorithm without hyperparameters. The coefficients of determination for grid search were higher for the no tuning algorithm (0.9) than grid search (0.887).

#### Polynomial regression

The 1st-degree polynomial had the highest correlation coefficient viz. 0.642. The coefficients of determination for the mean of the regressions and the best equation were 0.545 and 0.546 respectively.

#### XGBoost

A high correlation coefficient for the testing dataset was found for the XGBoost algorithm viz. 0.915. Low error values of prediction were also seen for this algorithm. The R^2^ values were equal to 0.88.

#### K nearest neighbors

For the breeding values, the k nearest neighbor algorithm was able to predict the breeding values with a correlation of 0.781 with the test dataset. The n neighbors arrived at using hyperparameter tuning were 9. The R^2^ value for the same was 0.635.

#### MARS

The correlation coefficient between the predicted and true values was found to be 0.746 while applying multivariate adaptive regression splines.

#### Algorithm ranking

The training and testing results of various algorithms are given in Table 5. Tree-based algorithms gave the best results with the random forest outperforming the rest by a small margin. Among these, random forests had the highest correlation coefficients (Table 6).

**Table 5:** Training and testing results of various algorithms.

	Support vector regression		Reg. trees	Random forests		Gradient boost		Polynm. reg		XGBOOST	
		Grid Search		Grid search	Random Search		Grid search	Mean	Best		
Hyper parameters	Kernel: rbf	C:6 Gamma:0.0001 Kernel: rbf	Default	Bootstrap: True max depth: 15 max features: auto n estimators: 20	n estimators: 23 max-features: auto max depth: 10 bootstrap: True	Default	Learning rate: 0.0 max depth: 4 n estimators: 2000 random state:1 subsample: 0.75	Default		Colsample-bytree: 0.7 Learning rate: 0.01 Max depth: 7 Min child weight: 1 n estimators: 1000 Objective: Sq. error Subsample: 0.7	Default
**Evaluation metrics**											
Test RMSE	0.608	0.695	0.496	0.384	0.383	0.388	0.39	0.642	0.649	0.392	0.645
Test MAE	0.685	0.74	0.597	0.524	0.53	0.531	0.537	0.545	0.546	0.537	0.705
Test r	0.777	0.699	0.869	0.917	0.917	0.915	0.912	0.705	0.706	0.915	0.746

“Default” suggests that the default hyperparameters of the algorithm were used for training the data. No hyperparameter tuning was done for the algorithms. (Reg. Trees = Regression Trees; Polynom. Reg. = Polynomial Regression). “rbf” = radial bases function.

**Table 6:** Ranking of algorithms for the prediction of breeding values.

Rank	Algorithm	Correlation coefficient (test)
1	Random forests	0.917
2	Gradient boosting algorithm	0.915
3	XGBoost algorithm	0.915
4	Classification and regression trees	0.869
5	Artificial neural networks	0.852
6	K nearest neighbours	0.781
7	Support vector machines	0.777
8	Principal component regression	0.746
9	MARS algorithm	0.746
10	Bayesian ridge regression	0.742
11	Polynomial regression	0.742

## Discussion

The values for coefficients of determination in our study were moderate to high for all models. High R^2^ values of 0.988, 0.929, and 0.976 using various machine learning approaches were also stated by Huma and Iqbal^6^ which correspond with the results obtained in the present study and indicate that machine learning approaches are quite effective in making animal-centric predictions. Valsalan et al.^7^ also used principal component analysis in Malabari goats to arrive at the growth performances and found the model obtained to have a coefficient of determination (R^2^) value equal to 74% which is similar to the result obtained in this study. A tenfold cross-validation approach was reported to train the best model by Huma and Iqbal^6^ which also correlated with the data split used in this study that was heuristically determined and any further increase in the validation dataset did not improve the results further.

Our results also indicate that PCA eliminated all multicollinearity in the dataset. This has also been established in literature by several authors^9,10^. PCA the present study was useful in allowing for a better understanding of the correlations among the traits at the same time, ensuring that feature reduction was achieved as was also stated by^9^.

A correlation coefficient of 0.658 was reported by Solberg et al.^11^ for the model to predict breeding values between true breeding values using PCR which is lower than the result of 0.746 as reported by us. Du et al.^12^ also endorsed the use of PCR in breeding value prediction in their study.

In the regression analysis for the breeding value dataset, seven features explained nearly 95% of the variance and is hence an effective technique for dimensionality reduction for large datasets. Pinto et al.^9^ also reported that the first five principal components explained nearly 93.3% of the variation, and the first component alone explained about 66%. The results obtained by Valsalan et al.^7^ also indicate that the first two components accounted for a high variance with an R^2^ value equal to 0.74. Khan et al.^13^ also reported the first two principal components to show maximum variance (61.86% and 26.14%). The components explaining a majority of the variance can be used for selection and breeding, especially for the construction of selection indices^14^.

The prediction equations derived for ordinary least squares had a moderate coefficient of determination. Such a moderate performance was also reported by Moser et al.^15^ who also found least-squares to not outperform other machine learning algorithms in their study to predict the breeding values of dairy cattle. Ordinary least squares regression has, however, been reported to give unbiased results with low variance as compared to many nonlinear models^16^. Ordinary least squares has been a popular technique in biometrical genetics for many decades.

The model predictions for ridge regression were similar to Bayesian ridge regression but the Bayesian models gave slightly better predictions. da Silva et al.^17^ also compared Bayes models to report that Bayesian ridge regression performed best for predicting breeding values. The use of penalties in the model for multiple predictors in the regression also makes it an effective technique^18^. The bottom-up approach of the Bayesian method which starts with priors has been reported to give robust results. The R^2^ value of the PCR model in this study was only marginally better than the Bayesian ridge regression model though Bayesian models have been seen to outperform OLS^19^.

Their study on the breeding values prediction using machine learning^20^, however, demonstrated a better predictive accuracy of ridge regression and Szyndler-Nędza et al.^21^ reported the regression model to perform better than the machine learning model for the prediction of carcass meat percentage which may be due to the less complexity of the problem at hand. Whittaker et al.^22^ proved the ridge regression model to be efficient to improve the mean response to selection and reduce the variability of the selection response. da Silva et al.^17^ used multiple Bayesian models for making genomic breeding values predictions and among them, the Bayesian ridge regression model had the lowest mean error value which is different from the results obtained in this study. A higher correlation of 0.90 between BV and predicted BV, using the Bayesian technique was observed for the prediction of BVs in the Harnali breed of sheep by Bangar et al.^23^.

Out of the models, model 12 had the highest correlation but a much higher correlation (0.92). A very high correlation was reported by Shahinfar et al.^24^ for the breeding values prediction in dairy cattle as well as Lopes et al.^25^ for the genomic. Artificial Neural Networks are being used in all spheres of biological sciences today. In our study, however, the tree-based algorithms outperformed ANNs. One contributing reason to this may be that the tree-based methods are deterministic and not probabilistic and thus perform well on structured data and their outperforming Bayesian methods may be justified.

A higher correlation of 0.89 between BV and predicted BV, using ANN was observed for breeding values prediction in Harnali breed sheep by Bangar et al.^23^. Our results correlate well with the reports of Ghotbaldini et al.^26^ for the breeding values prediction in Kermani sheep who used two ANN models to arrive at the correlation coefficients of 0.703 and 0.864 for them. The results obtained in this study are also consistent with the findings of other researchers in the areas of ANN application in animal science^8,27^.

The activation function called swish proved to achieve the best model convergence in artificial neural networks. Ramachandran et al.^28^ in their results, also found swish to consistently either match or outperform ReLU on deep neural networks. Swish function possesses strong regularization which is especially important for functions with negative values. Like our results, a low learning rate was similarly found to yield better results than a higher learning rate by Brownlee^29^ because large learning rates often lead to unstable training and may sometimes cause the neural network to never actually converge because the weights oscillate on the learning curve. Crump et al.^30^ used genetic algorithms for the estimation of molecular breeding values and showed that the correlation coefficients between actual and predicted values ranged from 0.66 to 0.79.

The dataset with default parameters could predict the test labels with a higher correlation than the grid search algorithm. The Gaussian process analysis of hyperparameter functions has revealed that all the hyperparameters do not matter in all machine learning algorithms and their importance depends on the type of search problem at hand. Due to this, the search sometimes does not produce the best solution.

SVR was reported to give the highest accuracy compared to many other machine learning methods for the breeding values prediction in dairy bulls by Moser et al.^15^. Ogutu et al.^18^ however reported a low correlation of SVR of 0.503 for genomic breeding value prediction and reported a value closer to the present study of 0.797. The rbf kernel gave better prediction results than the linear kernel. Long et al.^31^ also reported an improvement in correlation from 0.689 for the linear kernel to 0.700 for the rbf kernel. The better performance of the rbf kernel also indicates a nonlinear dependency of breeding values on the independent features.

For regression trees and random forests, the random search algorithm for the estimation of hyperparameters showed better model convergence compared to grid search. However, a lower correlation than the results in the present study between the predicted and observed trait responses was stated by Sarkar *et al.*^32^ viz 0.591 and 0.431 for random forests, and ridge regression, respectively. They also implied the superiority of random forests over ridge regression techniques in genomic prediction like our present study. Among these results, the random forests algorithm had the highest correlation coefficients, however, Neves et al.^33^ compared random forests and SVM for the genomic evaluation of a population of mice and did not observe any significant differences between the two methods. Sant’Ana et al.^34^ used eight regression-based machine learning techniques and found that the random forest regressor obtained R^2^ values of 0.687 and MAE of 3.099 suggesting that the model used in this study converged better than Sant’Ana et al.^34^ for the same model.

The 1st-degree polynomial predicted the breeding values with better accuracy than higher degrees. This also took the least amount of time to train. The popularity of linear models in the breeding values prediction also validates the results attained in this study.

A high correlation coefficient for the testing dataset was found for the XGBoost algorithm. Gradient boost gave higher correlation values than most other algorithms in the present study. Ogutu et al.^18^ who compared boosting, RF, SVM, and ridge regression BLUP (RRBLUP) also reported that accuracy was the highest for the boosting algorithm. Boosting algorithms are much greedier regarding decreasing the training error compared with SVM, which results in higher prediction accuracy, though this can reflect in longer computational time which was also seen in the present study. This is due to this strategy that boosting algorithms tend to have a lower training error as was also seen in the present study. Moreover, González-Recio and Forni^35^ compared multiple algorithms to report that boosting outperformed random forests, which is not in agreement with the present results. However, not unlike the results obtained in the present study, random forest and gradient boost reportedly consistently surpassed the XGBoost in the prediction accuracy^36^.

The K nearest neighbors’ algorithm was able to predict the breeding values with a correlation of 0.781 with the test dataset. The correlation coefficient between the predicted and true values was found to be 0.75 with a coefficient of determination of 0.557 while Aksoy et al.^37^ reported a much higher coefficient of determination of 0.968. They also reported that the MARS algorithm had greater predictive accuracy compared to the multiple regression analysis. The superiority of the MARS algorithm was reported in cattle^38^ (Aytekin et al.). Eyduran et al.^39^ obtained a lower R^2^ of 0.75 in the OLS for the prediction in goats. Also, Eyduran et al.^39^ found two ANN algorithms that they tested to be much inferior to those obtained for MARS. Similarly, Aytekin et al.^38^, Celik et al.^40^, and Ertürk *et al.*^41^ also highlighted the superiority of MARS.

The ML techniques used in the present study predicted the values that were derived using BLUP (Best Linear Unbiased Prediction). These were used as labels to evaluate how close the ML techniques would be to these values so that their convergence could effectively be tested. BLUP breeding values are standardized values that are proclaimed the world over for breeding values prediction with high accuracy^18^ and therefore act as standard values for any further research. True breeding values cannot be directly measured using phenotypic data^42^ and hence one must rely on EBV which has the highest accuracy. EBV or estimated breeding value is based on the information obtained from observed phenotypes. The addition of information from additional sources e.g., relatives and pedigree take the estimated values closer to the true breeding values by increasing their accuracy. The BLUP procedure combines all this information optimally and automatically^1^.

Comparison with any standard technique (use of controls) is a norm. This study used predicted breeding values instead of true values in most cases because the underlying relationships between features of true and predicted labels are the same and they are considered to be accurate.

BLUP breeding value estimations are cumbersome and extremely difficult for people with little know-how of animal breeding due to which it is hardly ever performed on farms, especially in developing countries. Therefore, if the technique is performed only once and the labels are subsequently used for training a model, that model can be deployed and used multiple times without any burden on computational resources.

Thus, the foundational research could help in handling the huge amount of data on farms especially as farm automation becomes a norm. The present research, therefore, used breeding values as labels to evaluate many techniques and find the ones that work best for sheep data. Also, the input data will always differ across farms, species, and even years which is a research limitation, but a robust technique with a larger and across-farm dataset would help in mitigating this to a large extent. The evaluation of unsupervised machine learning techniques and reinforcement learning could possibly help in overcoming this limitation as well.

## Conclusion

Globally, ML approaches are transforming animal genetics and this research was conducted to explore various techniques that could potentially impact the selection strategies on farms, especially in developing countries. A total of 12 reusable and deployable models were successfully developed for the prediction of breeding values. Most of the trained models had high prediction ability. Such models if adopted for the prediction of breeding values on the farms could help in the effective and timely selection of animals, especially in developing countries where selection is mostly intuition-based. The developed models are computationally much less expensive than the conventional methods and therefore have good prospects in future breeding strategies.

## Recommendations

The adoption of deployable ML models for the scientific selection of animals could help in the genetic improvement of animals. Machine learning could also be useful for managing other aspects of animal farming eg. early prediction of body weight, disease prediction, etc. The use of data spanning data across farms as well as the inclusion of genomic data can help in the development of models that can be used in diverse scenarios and farms.

## Methods

**Figure 2:**
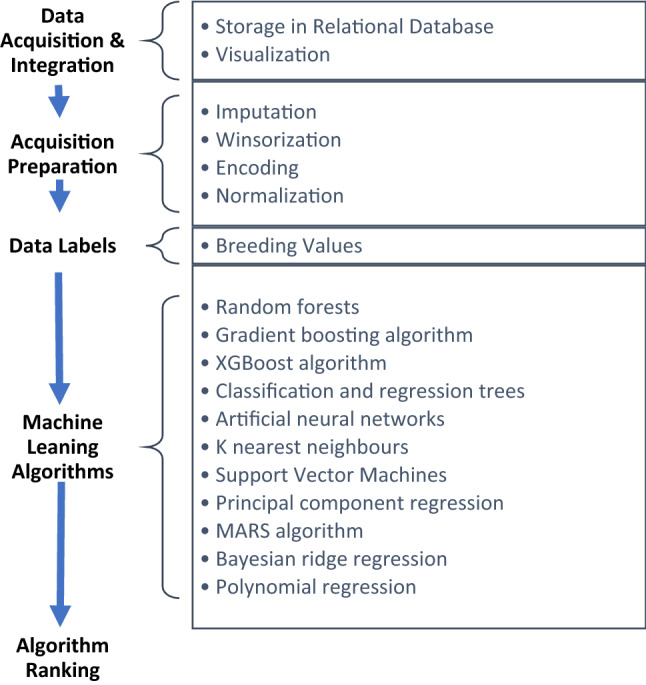
Brief research framework for the present study.

A brief research framework for the present study is presented in Fig. 2.

### Data collection

Data for the Corriedale breed of sheep for the last 52 years (1969–2021) was collected from the university sheep farm, SKUAST-K. The total number of data points available for the study was 76,835 with 18 features. The features included body weights at various ages of all animals under study, their pedigree, and other relevant features like sex, birth year, season, etc. Initially, the raw data was manually cleaned. Rows with missing values were treated as MAR (missing at random) values. Rows with too many missing values were removed altogether. Data imputation for the current dataset was done iteratively in Python^43^ based on multivariate imputation by chained equations (MICE). To handle the outliers in the dataset, the winsorization technique was used and the maximum winsorization limit was set at 99%^44^.

### Data preparation

The data was appropriately encoded before training the model. The least-squares means were used for a model with sex and the year of birth as fixed and these were used instead of label encoding year and sex. Label encoding years would have reduced the accuracy of the model under real-life situations. The dataset was also normalized. Pair plots in Python were used to check for multicollinearity.

### Input variable selection and labels

The final features/ input variables used for training the feature scores were determined heuristically and using the feature selection method. Through feature selection, an optimal feature subset was selected centered on the one that optimized the scoring function. The criterion set for the input variables to be used in all the machine learning approaches was determined based on feature scores. The input variables were kept constant across all ML methods to eliminate any bias caused by the uneven number of features/ input variables during training.

The EBVs (Estimated Breeding Values) for 12-month weights^1^ were derived and used as labels for training the data. BLUP animal model was used for the estimation of breeding values^45^. In this model, the fixed effects used were sex, year, and the random effects of the animal were used. Smart Sheep Breeder, a tool developed at SKUAST-K^46^ was used for the purpose. The model in the matrix and mixed model solutions are given below^47^:

1 $${\text{Y}} = {\text{Xb}} + {\text{Zu}} + {\text{e}}$$

2 $$\left[ {\begin{array}{*{20}c} {X^{\prime}X} & {X^{\prime}Z} \\ {Z^{\prime}X} & {Z^{\prime}Z + \lambda {\text{A}}^{ - 1} } \\ \end{array} } \right] \left[ {\begin{array}{*{20}c} b \\ u \\ \end{array} } \right] = \left[ {\begin{array}{*{20}c} {X^{\prime}y} \\ {Z^{\prime}y} \\ \end{array} } \right]$$

where, Y = Selected trait, b = Fixed vector for different non-genetic factors assumed to influence the traits, u = Random vector for breeding values of sires (to be predicted), e = Random error, X, Z = Incidence matrices, λ = (4-h^2^ )/h^2^, h^2^ = Heritability (estimated using animal model)^48^, A = Numerator Relationship Matrix. In this model, the fixed effects used were sex, year, and the random effects of the animal were used. Body weights at 12 month age were used as labels.

### Machine learning methods

Various machine learning algorithms were compared in this study. These included the following:

1. Principal component regression^49^: principal component analysis (PCR) for regression as a regularized shrinkage estimator was used. The principal components of the explanatory variables obtained from principal component analysis (PCA) were used as regressors. The principal components explaining most of the variance were used as features for training the dataset.
2. Ordinary least squares^50^: a technique for estimation of linear regression coefficients to minimize error between the actual and predicted values was used. It was aimed, through this technique, to minimize the sum of squared residuals between the actual and predicted values.
3. Bayesian ridge regression^43^: This technique was employed to evaluate whether output or response ‘y’ drawn from a probability distribution rather than a single value would train the model better than the others. The probabilistic model estimates of the regression problem were derived using this technique. The prior for the coefficient w was given by spherical Gaussian. Using this regression method, the L2 regularization was tested which is effective for multicollinearity^43^. The cost function in this method used a lambda term for penalty to reduce the model complexity, shrink the parameters to arrive at unbiased estimates.
4. Artificial neural networks^51^: Machine Learning technique inspired by biological neurons for finding optimum solutions to myriad problems. A typical neural network is a collection of connected units/nodes called artificial neurons^52^. The connection between neurons resembling synapses in a biological brain. Real numbers are transmitted as signals between neurons and the output of every neuron is computed by a non-linearity applied on the sum of its inputs. Neurons are aggregated into layers and as the number of layers increase, a dense neural network is formed.
5. Support vector machines^53^: supervised machine learning algorithm (SVM) for solving group classification problems or for regression analysis. SVM creates a maximum-margin hyperplane lying in a transformed input space to maximize the distance to the nearest cleanly split examples. The hyperplane solution parameters are derived from a quadratic optimization problem.
6. Classification and regression trees algorithm (CART)^54^: This algorithm builds a decision tree based on Gini’s impurity index to arrive at a final decision. In such decision trees each fork represents a decision causing a split in a predictor variable and each end node arrives at a prediction for the target variable.
7. Random forests^55^: an ensemble learning method that constructs many decision trees at training time to arrive at the most optimum solution. The mean or average prediction of all such trees is used as the final output for regression tasks.
8. Gradient boosting^56^: it uses an ensemble of many weak prediction decision trees, and the model is built in a stage-wise fashion. Generalizing other boosting methods, A gradient-boosting algorithms-built trees stage-wise by allowing the optimization of an arbitrary differentiable loss function.
9. Polynomial regression^57^: the relationship between independent and dependent variables is shown as the n^th^ degree polynomial. This regression technique offers an advantage that it fits a nonlinear relationship between x and y, which is denoted as E(y |x).
10. XGBoost^58^: a decision-tree-based ensemble algorithm using a gradient boosting framework for finding optimum solutions. The primary features of this technique include penalization of trees, extra randomization parameter, proportional shrinking of leaf nodes and newton boosting
11. K Nearest Neighbours^59^: a non-parametric learning classifier using proximity for making predictions about data points. This algorithm works off the simple assumption that points that are similar would be found close to each other. For regression problems, the average the k nearest neighbours is used as the prediction.
12. MARS^60^: finds many simple linear functions and aggregates them to find the best fitting curve for the data. In other words, Multivariate Adaptive Regression Splines combine a few linear functions using “hinges.” into an aggregate equation for making predictions in situations where linear regression or polynomial regression would not work.

### Model development

Models were developed and optimized either heuristically or using search algorithms or a combination of both. The details of each ML method are given below.

For optimizing the ANN models, hyperparameter optimization was attempted by the *Ax Client*^9^ for hyperparameter optimization and heuristic tuning was done as well. The optimization algorithm for included iterations = 2000, learning rate options = 0.001, 0.5, dropout rates = 0.01, 0.9, hidden layers numbers = 1, 10, neurons per layer = 1 to 400, batch sizes = 8, 10, 16, 20, 30, activation functions = 'tanh', 'LeakyReLU', 'ReLU', 'sigmoid', and Optimizer = 'adam', 'rms', 'sgd', 'RAdam'. 3 models were also created based on the number of iterations (500 for model 1, 1000 for model 2, and 2000 for model 3).

For support vector regression, for the hyperparameter tuning, a grid search in *sklearn* was performed. The grid search parameters for breeding values were param grid (c) =  − 1, 0.01, 0.1, 1, 6, 8, 10, 20, 50, 60, 100, 1000, gamma = 1e−4, 1e−3, 1, 0.1, 0.01, 0.001, 0.0001, 0.0009, kernel = 'rbf', 'sigmoid'.

Both grid and random search were attempted for regression tree hyperparameter tuning.

Hyperparameter tuning for random forests were bootstrap = True, max depth = 5, 10, 20, 15, 30, None, max features = 'auto', 'log2', n estimators = 5–13 and15, 20.

Up to 10 degrees of polynomials were tested for the polynomial regression. Each evaluation was attempted 6 times for all the datasets.

The gradient boost hyperparameters (grid search) were n estimators = 500,1000,2000, learning rate = 0.0001,0.001,0.01,0.1, max depth = 1,2,4, subsample = 0.5,0.75,1, random state = 1.

Hyperparameter tuning for XGBoost included learning rate = 0.001, 0.01, 0.05, 0.1, max depth = 3, 5, 7, 10, 20, min child weight = 1, 3, 5, subsample = 0.5, 0.7, colsample by tree = 0.5, 0.7, n estimators = 50, 100, 200, 500, 1000, objective = 'reg: squarederror'.

Grid search was employed for arriving at the best n-neighbors for KNN which were specified in a tuple as^2–8,17,44^.

MARS was used to fit the training data of all three datasets K fold cross-validation. The number of splits used was 10 and the number of repeats was equal to 3. TensorFlow Serving was used for the optimized models.

### Statistical metrics

The data was split into training and testing, and the optimal train test split was heuristically determined. The following percentages of data were used for constructing the model for most algorithms: testing data = 10% of the dataset, training data = 90% of the dataset, and validation data = 10% of training data.

For evaluating the models, the scoring criteria employed were mean squared error or MSE (Eq. 3), mean absolute error or MAE (Eq. 4), coefficient of determination or R^2^ (Eq. 5), and correlation coefficient or r (Eq. 6).

3 $${\text{MSE}} = \left( {1/{\text{n}}} \right) * \Sigma \;\left( {{\text{yi}} - {\text{xi}}} \right)^{2}$$

4 $${\text{MAE}} = \left( {1/{\text{n}}} \right) * \sum \left| {{\text{yi}} - {\text{xi}}} \right|^{2}$$

5 $${\text{R}}^{2} = {\text{r}}^{2}$$

6 $${\text{r}} = {{\Sigma \;\left[ {\left( {{\text{xi}} - {\text{xm}}} \right) * \left( {{\text{yi}} - {\text{ym}}} \right)} \right]} \mathord{\left/ {\vphantom {{\Sigma \;\left[ {\left( {{\text{xi}} - {\text{xm}}} \right) * \left( {{\text{yi}} - {\text{ym}}} \right)} \right]} {\surd \left[ {\Sigma \;\left( {{\text{xi}} - {\text{xm}}} \right)^{2} * \Sigma \;\left( {{\text{yi}} - {\text{ym}}} \right)^{2} } \right]}}} \right. \kern-\nulldelimiterspace} {\surd \left[ {\Sigma \;\left( {{\text{xi}} - {\text{xm}}} \right)^{2} * \Sigma \;\left( {{\text{yi}} - {\text{ym}}} \right)^{2} } \right]}}$$

where: yi = actual value for the ith observation, xi: calculated value for the ith observation and n: Total number of observations.

### Ethics declarations

This work is based on retrospective data and ethics declaration was not applicable in this study.

## Data Availability

The datasets generated and/or analyzed during the current study are not publicly available because permission is required from competent authority at the University but are available from the corresponding author upon reasonable request.
